# Supporting independence at home for people living with dementia: a qualitative ethnographic study of homecare

**DOI:** 10.1007/s00127-021-02084-y

**Published:** 2021-04-24

**Authors:** Monica Leverton, Alexandra Burton, Jules Beresford-Dent, Penny Rapaport, Jill Manthorpe, Ignacia Azocar, Clarissa Giebel, Kathryn Lord, Claudia Cooper

**Affiliations:** 1 UCL Division of Psychiatry, 6th Floor Maple House (Wing A), 149 Tottenham Court Road, London, W1T 7NF UK; 2 Centre for Applied Dementia Studies, University of Bradford, Bradford, UK; 3 NIHR Policy Research Unit on Health and Social Care Workforce, King’s College London, London, UK; 4 Department of Primary Care and Mental Health, University of Liverpool, Liverpool, UK; 5 NIHR Applied Research Collaboration North West Coast, Liverpool, UK

**Keywords:** Dementia, Homecare, Domiciliary care, Independence, Qualitative research, Ethnography

## Abstract

**Purpose:**

The aim of this ethnographic study was to investigate how homecare workers support or inhibit independence in people living with dementia.

**Methods:**

We undertook 100 h of participant observations with homecare workers (*n* = 16) supporting people living with dementia (*n* = 17); and 82 qualitative interviews with people living with dementia (*n* = 11), family carers (*n* = 22), homecare managers and support staff (*n* = 11), homecare workers (*n* = 19) and health and social care professionals (*n* = 19). We triangulated data and analysed findings thematically.

**Results:**

We developed three themes: (1) independence and the home environment, highlighting ongoing negotiations between familiarity, suitability and safety for care; (2) independence and identity, exploring how homecare workers’ understanding of their clients’ identity can enable active participation in tasks and meaningful choices; and (3) independence and empowerment, considering the important position of homecare workers to advocate for clients living with dementia while navigating authoritative power amongst proxy decision-makers.

**Conclusion:**

We consider that person-centred care should also be home-centred, respecting the client’s home as an extension of self. Homecare workers can use their understanding of clients’ identities, alongside skills in providing choice and developing relationships of interdependence to engage clients in everyday tasks. Homecare workers are well placed to advocate for their client’s voice within the care network, although their ability to do so is limited by their position within power structures.

## Introduction

Independence, considered central to active ageing, is defined by the World Health Organisation [1] as the ability to function in daily life with no or little help from others. Globally, dementia affects over 46 million people and is a leading cause of disability [2]. Living at home for as long as possible is an important manifestation of independence [3], although independence for people living with dementia may be more usefully conceptualised as the ability to manage life with ‘some degree of independence’ [4, 5]. Recent models of living independently with dementia acknowledge a tension between independence as an expression of full autonomy and the interdependence that can enable people with dementia to live longer at home [6]. Two-thirds of people diagnosed with dementia in the UK live in their own homes [7] often supported by family, friends, neighbours and care services.

Sixty percent of the UK homecare workforce work with people living with dementia [8] providing personal, basic nursing care and domestic support. Most homecare agencies explicitly adopt person-centred care values, which can be challenged by the task-orientated nature of care planning and delivery in an under-resourced sector. Person-centred dementia care considers individuals within their social context [9] and focuses on valuing the individual, promoting independence and functional abilities [10].

Few studies have included people living with dementia in research about their care. In one such study, people living with dementia prioritised independence (including opportunities for meaningful activities), self-management of dementia symptoms and quality of life [11]. In contrast, family members, who are often substituted as a proxy, prioritise their relative’s safety [12, 13]. We used ethnographic observations and qualitative interviews to answer our exploratory research question: how can homecare workers’ support or inhibit independence in their clients living with dementia? We sought to capture a wide range of stakeholder perspectives, from people living with dementia at varying stages of the condition, family carers, health and social care professionals (including general practitioners, social workers, and commissioners) and homecare staff. This work informed the development of the NIDUS-Professional training and support programme to enhance homecare workers’ understanding and implementation of person-centred dementia care [14].

## Methods

### Study design

This qualitative study was nested within the ‘New Interventions for Independence in Dementia Study’ (NIDUS) [ISRCTN15757555].

### Ethical considerations

London (Camden and King’s Cross) National Research Ethics Committee approved the study (reference: 17/LO/1713) in November 2017. We interviewed participants with capacity to consent, and people living with dementia without capacity to take part in research were included in participant observations, following the Mental Capacity Act (England & Wales) 2005 provisions to identify personal consultees who provided written consent. We obtained written informed consent from all participants with capacity to consent.

### Sampling and recruitment

For the qualitative interviews, we purposively recruited people diagnosed with dementia and family carers from three geographically diverse National Health Service (NHS) memory services, commercial homecare agencies, an Alzheimer’s Society Experts by Experience group and Twitter. Health and social care professionals were recruited via UCL, NHS memory services and local authorities. We recruited homecare managers, support staff and homecare workers from urban and semi-rural/rural homecare agencies. Purposive sampling ensured participant diversity in age, ethnicity, role (health and social care professionals and homecare staff), relationship to the person with dementia (family carers), homecare service experience (people living with dementia) and shift-pattern and client-type (homecare workers).

For the participant observations, we included homecare agencies whose staff had participated in our qualitative interviews and recruited additional agencies to observe care provision with both private and/or local authority funded clients from agencies across urban and semi-rural locations. Homecare workers planning to leave the agency within the next 6 months were excluded to ensure we could complete data collection.

### Data collection

We undertook: 1) semi-structured qualitative interviews (March 2018–September 2018); and 2) participant observations (August 2018–March 2019).

Qualitative interviews took place in locations convenient for participants. People living with dementia and family carer dyads were interviewed together if preferred. Interviews were guided by a semi-structured topic guide (Appendix A) and lasted around 1 hour; they were audio-recorded and transcribed verbatim. Participants were offered a £20 voucher for their time. Recruitment ceased when saturation was reached [15].

The participant observations were guided by our earlier methodological review of observational studies of homecare [16]. Three researcher-observers (ML, AB and JBD) from psychology and sociology backgrounds conducted naturalistic observations of homecare workers, guided by a semi-structured observation guide (Appendix B) intended to enhance consistency of fieldnotes across observers and prompt reflexivity. Researcher-observers aimed to minimise impact on homecare visits but did engage conversationally with participants, using an ethno-interview technique to enrich fieldnotes [17]. After one or two familiarisation visits to build rapport, we observed up to five further visits, taking brief fieldnote ‘jottings’ which were completed after the visit [18]. Observations included short (i.e. 15-min personal care visit) and long visits (i.e. 3-h respite or ‘sitting service’), funded privately or by local authorities. Observations were capped at 2 hours to prevent participant burden and researcher fatigue. The researcher-observers met weekly to discuss their observations and reflective notes. The data collection process is detailed further in Appendix C.

### Data analysis

We adopted a critical realist lens [19] and thematically analysed transcribed interviews and observational data [20, 21], triangulating findings between sources. We used NVivo12 software to organise the data and performed an initial inductive analysis of each dataset. ML and two members of the research team independently coded 25% of the interviews across all participant groups to develop an initial coding framework. ML and other research team members coded 15% of observation fieldnotes from which ML developed a second coding framework. We drew on the ‘Following a thread’ methodology [22] to iteratively integrate the findings, exploring how codes from one dataset followed into the other, and vice versa, developing one interwoven framework. We then applied the framework to the remaining interviews and half of the observation fieldnotes, adding new codes to the framework until no new codes were found. The remaining fieldnotes were read in detail and compared against the framework to ensure verification, comprehension and completeness of the data [23]. Themes were refined and defined following discussions within the team. We also looked for divergences between the two datasets.

## Results

### Study participants

We interviewed 82 participants: 11 people living with dementia (PLwD), 22 family carers (FC), 11 homecare managers and support staff (HMSS), 19 homecare workers (HCW) and 19 health and social care professionals (HSCP). Health and social care professionals included Medical General or Assistant Practitioners, Psychologists, Social/Support workers, Local Authority Commissioners (funders), amongst other roles. We conducted 100-h of participant observations with 16 homecare workers and 17 clients living with dementia, across 6 homecare agencies. Where possible, we travelled with homecare workers between clients’ homes and observed them interacting with agency staff. Tables 1, 2, 3 for participant demographic information and (Table 4) for homecare agency information.

**Table 1:** Details of qualitative interview participants (homecare workers are presented in Table 2)

Characteristics	Category	Homecare managers & support staff *n* = *11*	People living with dementia *n* = *11*	Family carers *n* = *22*	Health & social care professionals *n* = *19*
		*n* (%) or *mean (SD)*	*n* (%) or *mean (SD)*	*n* (%) or *mean (SD)*	*n* (%) or *mean (SD)*
Age		*47.3 (9.5)*	*78.6 (7.8)*	*57.7 (14.3)*	*41.4 (10.9)*
Gender	Female	9 (81.8)	5 (45.5)	12 (54.5)	13 (68.4)
	Male	2 (18.2)	6 (54.5)	10 (45.5)	6 (31.6)
Ethnicity	White–British	7 (63.6)	8 (72.2)	9 (40.9)	9 (47.4)
	White–Irish	1 (9.0)	1 (9.1)	0 (0.0)	1 (5.3)
	White–Other	0 (0.0)	0 (0.0)	0 (0.0)	4 (21.1)
	Asian–Indian	0 (0.0)	0 (0.0)	6 (27.3)	2 (10.5)
	Asian–Bangladeshi	0 (0.0)	0 (0.0)	4 (18.2)	0 (0.0)
	Black/Black British–African	2 (18.2)	0 (0.0)	0 (0.0)	0 (0.0)
	Black/Black British–Caribbean	1 (9.0)	0 (0.0)	0 (0.0)	0 (0.0)
	Other	0 (0.0)	2 (18.2)	3 (13.6)	3 (15.8)

**Table 2:** Self-reported demographic information of homecare worker participants across the two methods of data collection: qualitative interviews and participant observations

	Homecare workers^a^: interview *n* =19	Homecare workers: observation *n* =16
	*n* (%) or *mean (SD)*	*n* (%) or *mean (SD)*
Age	*48.9 (12.9)*	*49.0 (5)*
Gender		
Female	16 (84.2)	16 (100)
Male	3 (15.8)	0 (0)
Ethnicity		
White British	15 (78.9)	12 (75)
White Other	1 (5.3)	1 (6)
Black/Black British Caribbean	1 (5.3)	1 (6)
Black/Black British African	1 (5.3)	2 (13)
Other	1 (5.3)	0 (0)
Contract type		
Employed on zero hours contract	3 (15.8)	5 (31)
Employment		
Working part time	8 (42.1)	5 (31)
Working full time	9 (47.4)	11 (69)
Other (e.g. varied shift patterns)	2 (10.5)	0 (0)
Years worked in social care		
6 months–1 year	3 (15.8)	3 (19)
1–3 years	2 (10.5)	4 (25)
3–5 years	4 (21.1)	1 (6)
5–10 years	5 (26.3)	4 (25)
More than 10 years	5 (26.3)	4 (25)
Years worked in current agency^a^		
Less than 6 months	2 (10.5)	1 (6)
6 months–1 year	4 (21.1)	3 (18)
1–3 years	5 (26.3)	7 (44)
3–5 years	3 (15.8)	2 (13)
5–10 years	3 (15.8)	2 (13)
More than 10 years	1 (5.3)	
Personal experience of dementia in family/friend		
Yes	9 (47.4)	6 (38)
No	10 (52.6)	10 (62)

^**a**^One homecare worker (interview only) was unable to specify length of time worked in current homecare agency

**Table 3:** Characteristics of clients living with dementia observed receiving homecare (*n* = 17)

Pseudonym	Age	Sex	Ethnicity	Living situation	Capacity to consent	Care funding	Scheduled visit duration	Requires support with
Betty	*Missing data*	Female	White British	Lives alone	Yes	Private	3 h	Medication management, meal preparation, prompt washing and support with dressing, domestic support, arranging and accessing appointments in the community and food shopping
Beverley	77	Female	White British	Lives with spouse	No	Private	1 h	All support delivered in bed: personal care, dressing, companionship and domestic support
Bonnie	84	Female	White British	Lives alone	Yes	Private	1.5–3 h	Personal care, meal preparation, medication management, domestic support and accessing the community
Belinda	82	Female	Black Caribbean	Lives alone	No	Local authority	30 min	Meal preparation, meal time companionship and medication management
Barbara	80	Female	White British	Lives with son	No	Local authority	30- 45 min	Getting out of bed, personal care, meal preparation, companionship during mealtime and medication management
Brian	61	Male	White British	Lives with spouse	No	Local authority	3 h (sitting service)	Respite for family carer, meal preparation and personal care
Beth	85	Female	White British	Lives with spouse	No	Local authority	15–30 min + 4 h respite visits twice weekly	All support delivered in bed: personal care, reposition and assess pressure areas, transfer using hoist and respite for family carer
Beatrice	96	Female	White British	Lives alone	No	Local authority	3 h shifts within 24 h care package	All personal care needs require support
Brenda	93	Female	White British	Lives alone	Yes	Local authority	30 min	Meal preparation
Benji	84	Male	White British	Lives with spouse	No	Local authority	30 min	Personal care, support with dressing and medication management
Bernice	89	Female	White British	Lives alone	Yes	Local authority	15 min	Meal preparation and medication management
Bridgette	94	Female	White British	Lives alone	Yes	Local authority	30 min	Meal preparation, medication management and domestic support
Boris	77	Male	British	Lives with spouse	No	Private	2 h	Personal care, meal preparation, companionship, mental stimulation and mobility support around home
Bara	98	Female	*Missing data*	Lives alone	No	Private	1 h	Personal care, support with dressing, meal preparation, administering medication and domestic support
Benita	88	Female	British	Lives alone	No	Private	1–5 h	Domestic support, companionship, accessing the community, arranging appointments, food shopping and pet care
Bryony	99	Female	White British	Lives alone	No	Local authority	30 min	Personal care, dressing, meal preparation, medication management and domestic support
Bea	89	Female	White British	Lives alone	No	Local authority	30 min	Meal preparation, medication management, domestic support and companionship

**Table 4:** Characteristics of homecare agencies participating in observations (*n* = 6)

Homecare agency	Location	Care Quality Commission (CQC) rating	Total number of clients (% of clients with dementia or memory problem)	Homecare workers on zero hours contract (% of all employed)	Client funding	Dementia-specific training offered	Homecare workers observed	Clients with dementia observed
1	London	Good	91 (39.5)	85 (100.0)	Private	Accredited training, offered quarterly or as needed	1	1
2	London	Good	150 (4.6)	90 (100.0)	Local authority	Non-accredited in-house training, offered at point of induction + yearly	2	2
3	South England	Good	28 (53.6)	1 (6.6)	Private	Non-accredited training at point of induction, some staff offered external advanced dementia training	3	2
4	South England	Good^a^	180 (45.0)	67 (95.7)	Private and local authority	Accredited training, offered yearly	5	7
5	North England	Outstanding	112 (62.5)	74 (93.7)	Private and local authority	Dementia awareness training at point of induction + accredited training offered to some staff	3	3
6	North England	Good	196 (31.6%)	120 (95.2%)	Private and local authority	Accredited in-house training, offered at point of induction + yearly	2	2

^a^CQC rating changed from ‘Good’ to ‘Requires Improvement’ at the start of the observation period

### Qualitative analysis

Interview participants are anonymously identified by abbreviations as above and ID number, while observation participants are pseudonymised: ‘A’ names for homecare workers, ‘B’ names for people with dementia and ‘C’ names for family carers.

We developed three themes capturing how homecare workers support or inhibit independence in clients living with dementia:

#### Theme 1—Independence and the home environment

All participant groups interviewed described *“being in their own home in their own surroundings”* (HCW19-interview) and the ability to *“move freely around their home, to do whatever it is they want to do”* (HCW17-interview) as key to maintaining independence. Our two subthemes explore how the home environment enabled independence through familiarity but could also compromise (1) the delivery of the care; and (2) client safety, requiring a balancing of risk and autonomy.

##### Subtheme 1: Familiarity versus adaptation

Delivering care in homes not designed as care environments often resulted in imperfect solutions which were necessary to enable essential care, but could inhibit clients’ access to parts of their home, and the independence the care sought to enable, as well as to homecare workers’ safety and wellbeing:

‘*Audrey tells me that they are unable to fit the wheelchair through the door frames so Beatrice mainly spends her time between the TV room and her bedroom next door.’ (Fieldnote).*

*‘Amy manoeuvres the hoist as Ava holds Beth’s legs still. Amy struggles to swiftly manoeuvre the hoist on the carpeted floor having to put physical effort into pushing it. Amy bumps into a table as she does this which jolts Beth.’ (Fieldnote).*

As clients’ homes often required adaptations to support independent living, the environment shifted from a personal space to one with visible signs of disability and care: disposable gloves, bathroom adaptations, living rooms converted into bedrooms, agency files and notes, and homecare workers’ possessions:

*‘Annie says that the dining table is used to leave messages to those involved in Benita’s care–e.g. a folder that bills are put into for the family, notes for other homecare workers, *etc*. Annie apologises to me that it looks messy.’ (Fieldnote).*

*‘There is a bike obstructing the hallway–Audrey tells me that one of the other homecare workers had left her bike in the house while she was on holiday. Beatrice had noticed a few times and asked for it to be taken out of her home.’ (Fieldnote).*

These changes and adaptations could be depersonalising and overshadow the familiarity of the home. The latter example suggests that Beatrice’s home was not always treated as a private space, and her requests for the bike to be removed were unheeded.

These adaptations, however, often enabled people with dementia to continue living in a familiar environment. We observed one client and his wife who had recently moved to a more accessible home with a wet-room equipped with rails. Here, we heard how the loss of familiarity of a known environment was frightening for the  person living with dementia:

‘Caroline informed me earlier that Brian finds the bathroom claustrophobic and they [FC and HCW] often have trouble getting him in there.’ (Fieldnote)

### Subtheme 2: Safety and risk

There was often uncertainty about whether the risks of remaining in the familiar, yet sometimes unsuitable environment were justified. One client’s house had steep stairs; which the homecare worker herself had reported feeling ‘unsteady’ on. This exacerbated the homecare worker’s concerns on visits where this client was slow to open the door or failed to answer when she arrived:

*‘The atmosphere felt tense while standing outside Bernice’s door as Alison attempted to gain entry. After several times ringing the front door, Alison asked the agency to call Bernice’s house phone which she did not answer. Alison sighed and said she didn’t think it was fair that Bernice lived on her own.’ (Fieldnote).*

Bernice also used a chair to barricade her front door overnight, while another client expressed feeling ‘vulnerable’ while waiting for his wife to return home after the homecare worker had left.

Risks associated with the home environment were a major concern for homecare workers and family carers, but all participants described needing to find a balance between managing risk and supporting independence:

*“I think she is entirely fed up with me, I think she sees me as like a jailer really. Which I'm not, I'm only trying to prevent her falling or helping her… It’s difficult to judge the line.” (FC32-interview).*

*“…you can only keep them as safe as you can. There will always be falls. I was always taught that if they’re not falling then they’re not living. Because they’re not moving around, you see?” (HCW28-interview).*

One response to managing risk was to restrict clients’ access to more risky or hazardous parts of the home:

*‘…Angela reminds Betty that her bedroom is now downstairs. Although jovial, Betty compares this to a dog being locked out of its room. Angela reminds Betty that her cousin Cliff had asked for this in order to keep her safe.’ (Fieldnote).*

#### Theme 2—Independence and identity

We saw and heard how homecare workers could use their often extensive knowledge about a client, their identity and wishes, to enable independence through offering meaningful choices and involvement in decision-making. Our two subthemes explore: (1) understanding and valuing the client’s identity and (2) mechanisms homecare workers used to help clients stay involved and making choices.

##### Subtheme 1: Understanding the client

Acknowledging the personhood of people living with dementia was a prerequisite to enabling independence:

“We all had talents before dementia; we don’t suddenly lose those talents overnight when we’ve got a diagnosis. And to remember that we were once a working person and a totally capable person. So to remember that there’s been a person there that still can do things.” (PLwD17-interview)

Being able to connect the past with clients’ present lives was important for homecare workers; to be able *“to see their clients… to have some understanding of where they have come from”* (FC11-interview). Care provided in clients’ homes, surrounded by their life memories and possessions facilitated this:

“We have a chap who… he was one of the first British soldiers to get captured and he spent the whole of the war in a prisoner of war camp. And he got out the photographs and he showed me… he could sit and talk for hours and hours and it obviously made him very happy… so it was always a good starting point for the homecare workers.” (HCM001–interview)

In some observations, there was a sense that the client’s identity and personhood were becoming challenged or lost, for example where homecare workers used infantilising language or behaviour. In the example below, the client was able to challenge this:

‘Amy tells Beth that she’s going to “give her little feety a wash now”, to which Beth exclaims “I don’t have a little feety, I have a foot!”.’ (Fieldnote)

##### Subtheme 2: Staying involved and making choices

We observed homecare workers supporting clients to actively participate in their care; for example, by simplifying or breaking down tasks. The examples below highlight the effect of different approaches taken by two different homecare workers with the same client:

*‘Amy told Beth they were going to wash her face. As she begins, Beth asks what she’s doing with her face and Amy explains again. Beth says that she’s not used to being washed and dressed by other people, she’s used to doing it herself and begins to cry.’ (Fieldnote).*

*‘Alice told Beth they were going to wash her hands and then asked Beth if she would like to try washing herself as it ‘might be good for her’ to do this… Beth was able to use the flannel to wash most of her upper body, while Alice provided step-by-step instruction to Beth which she was able to follow–e.g. “now use this hand and wash under this arm”, while touching each to guide Beth.’ (Fieldnote).*

In interviews, all participant groups highlighted the importance of involving people living with dementia in everyday tasks, even *“if they can’t do it in a way that we expect it”* (HCSS02-interview). Often, this involved establishing relationships of interdependence and making required adaptations:

*“I think the majority of people would still want to do things for themselves. So, it’s what you can advise the person, like if the person can’t tie their shoelaces anymore, then advise them and the family to get slip-on shoes.” (PLwD17-interview).*

Care approaches that enabled active participation in tasks and meaningful choice were observed to facilitate independence, but these could be overshadowed by time constraints, or a perceived obligation to provide the ‘right care’, particularly with clients whose abilities to participate in tasks and express choices were declining:

*‘Anya says to Belinda “go on eat your food”. Belinda is eating her cereal but slowly. She says to Belinda, “go on eat it”, she takes the spoon out of Belinda’s hand and stirs the cereal and says again “go on eat it”.’ (Fieldnote).*

‘Angela asks if Betty would like a drink and she chooses both squash and coffee. Angela says she has already had coffee, she can have a lemon tea later on instead.’ (Fieldnote)

#### Theme 3–Independence and empowerment

We saw how homecare workers advocated for their clients, ensuring the client’s voice remained central to the care they received. This was often enabled by homecare workers, family carers and the person living with dementia working in collaboration:

*“It’s a three-way street… Between the carer, the client and the family. As long as the family is wanting to put the input in, that in turn helps us, which then in turn allows us to do that bit extra for our client.” (HCW17-interview).*

When this three-way relationship worked well, it could strengthen the voice of the person living with dementia:

“I think there’s something about making sure the person with dementia has a voice, and not talking for them, even if their voice sometimes is very muddled.” (HSCP17-interview)

Most clients observed were not able to make all their own decisions about care, with family carers frequently substituting as decision-makers. Homecare workers often felt unsure of whether and how to challenge or question proxy decisions with which they disagreed, as, while privileging the client’s voice might be construed as supporting their independence, it could also be seen as directly challenging the family carer’s authority:

*‘In the hallway, Cameron tells Alyssa that Beth had just said she didn’t want to get out of bed today, but they should ignore that and get her in her chair. He added that if this was too problematic, the occupational therapist had said to just leave her in bed.’ (Fieldnote).*

*“I know you’ve got to respect the families’ choices as well, but I think the client’s choices are important and I think you’ve just got to do things for the better of the client for what is going to be more beneficial to them.” (HCW20-interview).*

There seemed a fine-line for homecare workers between providing support with *“a bit of diplomacy”* (HCW23-interview) and stepping out of place with family carers. One homecare worker who was observed taking on excessive responsibilities for her client at the request of family carers, was later asked to leave by the family for becoming too involved in the client’s home:

*‘Angela can no longer see Betty and must not return to her house to say goodbye; she is upset and concerned for Betty as she didn’t have a chance to tell her she wouldn’t be coming back.’ (Fieldnote).*

Though we only heard one perspective on this decision, the sudden dismissal *‘after providing such committed care to Betty for over two years’ (fieldnote)*, illustrates the insecurity of the homecare worker role. Other homecare workers described feeling powerless to influence their client’s living situation:

*‘Anna says that she fears Beatrice will not have the strength to hold herself up or walk after being in bed for one week. She says that the social services’ decision to keep Beatrice in bed has “completely taken everything away from her” and feels there is nothing they as homecare workers can do about it.’ (Fieldnote).*

Whilst many homecare workers felt they could draw on support from agency managers, they may still be hindered by the power dynamics involved in homecare and the insecure position of homecare workers within the client’s multidisciplinary care. This, in addition to the vulnerability of the role, may be a source of stress for homecare workers when they wish to advocate for what they believe to be in their client’s best interest.

## Discussion

### Main findings

In responding to our aim of exploring how homecare workers support or inhibit independence in people living with dementia, we observed the familiarity of home as an enabler, and explored how adaptations can facilitate necessary and often safer care, yet depersonalise the home. Homecare is delivered amongst clients’ life possessions, giving homecare workers insight into clients’ identities. Through familiarity with the client and their functional abilities, care approaches that enabled active participation in tasks, and collaborative working with clients and family carers in decision-making, we saw how homecare workers could advocate for the client’s ‘voice’. However, they could be unsure how to do so meaningfully with clients who have more advanced dementia, and whether and how to advocate with proxy decision-makers, amongst whom they could feel powerless.

### Practice implications

Cahill [24] stated that changes to facilitate homecare should not reduce autonomy, independence, dignity, nor heighten changes to the self for people living with dementia. In practice, adaptations can involve trade-offs between these values and facilitating care that enables independence [25, 26]. Our findings illustrate the importance of minimising environmental changes and considering their potential impact carefully. We posit that care that is person-centred need also be home-centred, respecting the client’s home as an extension of self. Reconstruction of the private home into a place of work has been discussed with other long-term care recipients [27]. We observed how homecare workers and the delivery of homecare could blur boundaries between the client’s private space and the homecare worker’s workplace; for example, where one homecare worker stored her bike in a client’s hallway whilst on holiday. This must be considered in the context of homecare, where homecare workers typically work in isolation and have no shared place of work [28].

Engaging clients living with dementia in roles and tasks associated with the self reaffirms their sense of self identity [29]. Our observation of a client living with dementia challenging a homecare worker who infantilised her, highlights the client’s awareness or ‘continuity’ of self [30]. NHS England’s Well Pathway for Dementia [31] and other national initiatives stress the importance of promoting independence for people living with dementia, and enabling meaningful care [32]. We observed that the extent to which this was possible was limited by time-constraints and how providing people living with more severe dementia with meaningful choices was particularly challenging.

Rønning [33] described the need to find a balance between independence, dependence and interdependence. Interdependence, its intricacies and complexities, was evident in our observations. Where it is possible for homecare workers to establish a relationship of interdependence with clients living with dementia and their family carers as a team, there may be associated benefits such as prolonged functional abilities, decision-making and acknowledgement of important social relationships [13, 34–37]. Participants highlighted the importance of relationships being negotiated in a way that enabled the client’s voice to be heard; our observations illustrated some of the frustrations experienced when the, at times, delicate support network that supported this interdependence was stressed or unbalanced. Scales and colleagues [38] argued that empowerment of care staff to provide flexible, person-centred care requires wider-level practice change beyond the current power constructs that influence dementia care. Many homecare workers know their clients living with dementia very well, and they may be in a unique position to advocate. Yet, in our observations, homecare workers often felt unable to do so. Powerlessness of care staff has been explored in residential settings, whereby organisational rules and demands often take precedence over person-centred care, resulting in some staff breaking rules to promote individualised care for residents [38–40]. Our findings extend this to show the vulnerability of homecare workers’ position and limited power within the structure of homecare (see also Manthorpe et al. [41]). Professionalisation of the workforce is a potential, yet widely debated solution to improve societal and professional status of the homecare workforce [38, 42, 43].

### Strengths and limitations

Our findings build on an important but under-researched area, exploring independence and homecare for people living with dementia. We represented diverse participant perspectives to explore our research question. Participant observations allowed us to capture the lived experience of homecare through the eyes of both the care provider and receiver; and the subjective experiences of people living with more severe dementia that are seldom researched.

Whilst we sought to represent diversity in CQC ratings of homecare providers, our sample consisted of agencies predominantly rated ‘Good’. This is reflective of providers in England: 80% were rated ‘Good’ in 2020 [44]. In our recruitment process, agency managers selected homecare workers to participate in the study which may have entailed bias in selection of the ‘best’ care workers to reflect the agency positively. To minimise this, we asked agency managers to ask all eligible homecare workers who supported clients living with dementia. Our study was limited by situations we were able to observe (i.e. we only observed day-time visits), as well as the potential for the Hawthorne effect on those observed [45]. We did not collect quantitative data so cannot quantify the severity of dementia or of neuropsychiatric symptoms, however, we explored the varied lived experiences of people living with dementia being observed within our qualitative analysis.

### Future research

Homecare for people living with dementia should continue to be explored through inclusive methods of data collection. Our study, in addition to previous research [16], found ethnographic participant observations were well-suited to capturing the lived-experiences of people living with dementia, including people who lack decision-making capacity, in the private setting of their home. Participant observations of care delivered in the home highlighted important considerations about the home environment itself and the way homecare workers interact with the home. The impact of the built environment on people living with dementia has been studied in residential settings [48], but less is known about the impact of the home environment on care provision.

## Conclusion

We propose that care that is person-centred is also home-centred, respecting the sanctity and familiarity of the client’s home as an extension of self. Homecare workers can support clients living with dementia to live at home as independently as possible by acknowledging and valuing their existing identity, facilitating involvement in tasks and decision-making, and working collaboratively with clients and family carers to make deliver care with the client’s ‘voice’ at the centre. Interdependence may be the more optimal concept to strive for with clients living with dementia, particularly with clients whose ability to express choices and make decisions is declining. Homecare workers could hold an important position in care networks, though their potential to achieve this is often limited by their position within power structures; we consider professionalisation of the workforce as a potential solution to address these issues.

## Figures and Tables

**Table 6:**
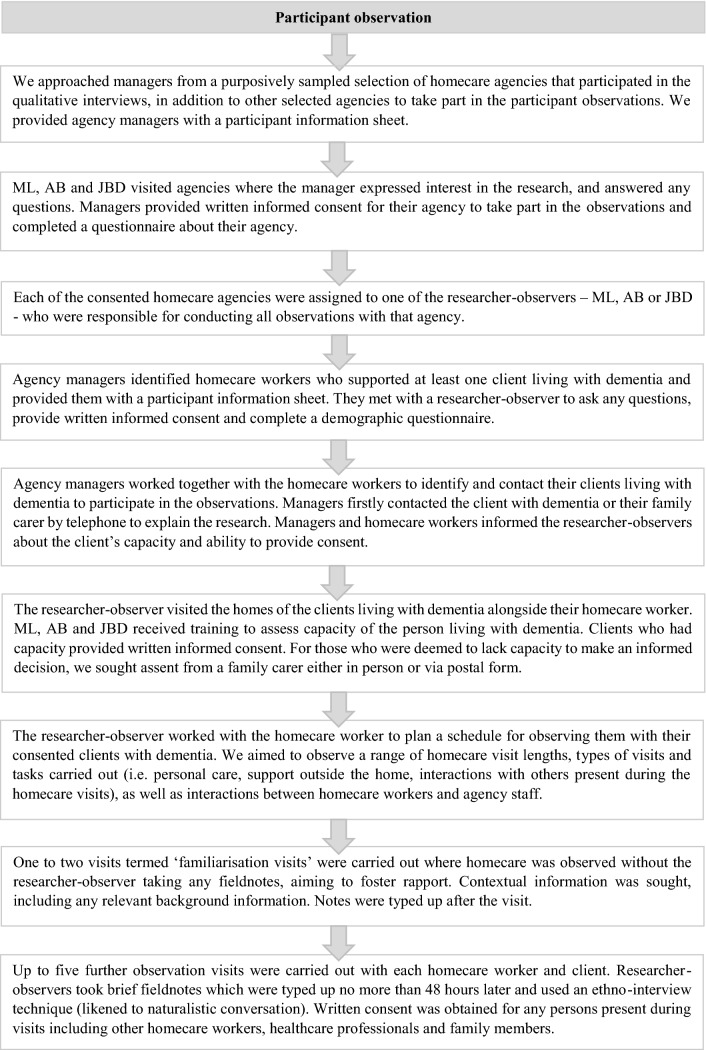
Data collection process of participant observations

## Data Availability

The qualitative data will not be deposited as it is potentially identifiable to participants, and, therefore, as is customary with qualitative datasets, will not be deposited in a public depository. However, we can potentially make data available to researchers who obtain appropriate consents from an ethics committee.

## Appendix A

(See Table 5).

**Table 5:** Semi-structured interview topic guide

Participant topic guide	Questions related to independence at home in people living with dementia
Family caregivers	To what extent is [the person you care for] currently able to live independently at home? What do you find independence means for the person you care for? What do you feel being independent looks like for them? Can you think of a time since [the person you care for] has had memory problems, when they have/have not been able to achieve or do something independently that has been important/difficult for them? What happened? What made it easier/harder? What makes it harder/easier for the person you care for to live independently at home?
Homecare staff and Health and Social Care Professionals	What do you find independence means for your clients with dementia? What do you feel being independent looks like for them? Can you think of a time when a client with dementia has been/has not been able to achieve or do something independently that has been particularly important/difficult for them? What happened? What makes it harder/easier for your clients with dementia to live independently at home?
Person Living with dementia	What do you do to live independently at home? Do you get any help from anyone else (paid carer/family member)? What do they do? What can make it harder/easier to stay independent?

## Semi-structured participant observation guide

### Guidance: areas of interest to keep in mind during the observation:

1. **A practical overview of the visit**
  - record the time at which the home carer arrives and leaves the client’s home
  - who is present
  - the environment in which the care is being delivered including physical layout, decor and cleanliness
  - “atmosphere” including general feelings about tension, is it welcoming, comfortable etc.
  - the tasks that are delivered

2. Interactions and responses of home carers with clients and others
  - interactions and responses between the home carer and the client with dementia
  - interactions and responses between the home carer and others who may be present
    o positive, negative and neutral interactions/responses
    o support of independence/choice/autonomy where possible
    o challenges to independence (e.g. symptoms of distress, refusal of care or risks) and responses to it where this occurs, and whether these strategies are effective in resolving distress and enabling necessary care to be given
    o emotional responses, strategies and resources used (e.g. practical, social, spiritual)
  - how client (including behaviour, language, ethnicity and culture), family carer, home carer and/or management and organisational factors impact on care provided
  - whether additional needs arise, either stated by the person with dementia, family carer or home carer and how these are managed.
  - references to client or family carer goals or priorities, how these emerge and how they are acknowledged or not.
  - general thoughts and feelings about the care being delivered and how the care provided enables or disables independence
  - how your presence as a non-participant observer may have influenced your observations

## Appendix C

(Table 6).
