# Correction to: Supporting independence at home for people living with dementia: a qualitative ethnographic study of homecare

**DOI:** 10.1007/s00127-021-02165-y

**Published:** 2021-09-04

**Authors:** Monica Leverton, Alexandra Burton, Jules Beresford-Dent, Penny Rapaport, Jill Manthorpe, Ignacia Azocar, Clarissa Giebel, Kathryn Lord, Claudia Cooper

**Affiliations:** 1 UCL Division of Psychiatry, 6th Floor Maple House (Wing A), 149 Tottenham Court Road, London, W1T 7NF UK; 2 Centre for Applied Dementia Studies, University of Bradford, Bradford, UK; 3 NIHR Policy Research Unit on Health and Social Care Workforce, King’s College London, London, UK; 4 Department of Primary Care and Mental Health, University of Liverpool, Liverpool, UK; 5 NIHR Applied Research Collaboration North West Coast, Liverpool, UK

## Correction to: Social Psychiatry and Psychiatric Epidemiology 10.1007/s00127-021-02084-y

The article “Supporting independence at home for people living with dementia: a qualitative ethnographic study of homecare”, written Monica Leverton, Alexandra Burton, Jules Beresford Dent, Penny Rapaport, Jill Manthorpe, Ignacia Azocar, Clarissa Giebel, Kathryn Lord, Claudia Cooper was originally published electronically on the publisher’s internet portal on 24 April 2021 without open access. With the author(s)’ decision to opt for Open Choice the copyright of the article changed on 9 September 2021 to © The Author(s) 2021 and the article is forthwith distributed under a Creative Commons Attribution 4.0 International License, which permits use, sharing, adaptation, distribution and reproduction in any medium or format, as long as you give appropriate credit to the original author(s) and the source, provide a link to the Creative Commons licence, and indicate if changes were made. The images or other third party material in this article are included in the article’s Creative Commons licence, unless indicated otherwise in a credit line to the material. If material is not included in the article’s Creative Commons licence and your intended use is not permitted by statutory regulation or exceeds the permitted use, you will need to obtain permission directly from the copyright holder. To view a copy of this licence, visit http://creativecommons.org/licenses/by/4.0. Open access funding enabled and organized by Projekt DEAL.

The original article was corrected.

